# Gypsum Cave Biofilm Communities are Strongly Influenced by Bat- And Arthropod-Related Fungi

**DOI:** 10.1007/s00248-024-02395-y

**Published:** 2024-06-03

**Authors:** Valme Jurado, Tamara Martin-Pozas, Angel Fernandez-Cortes, Jose Maria Calaforra, Sergio Sanchez-Moral, Cesareo Saiz-Jimenez

**Affiliations:** 1 Instituto de Recursos Naturales y Agrobiologia, IRNAS-CSIC, 41012 Sevilla, Spain; 2 Departamento de Biologia y Geologia, Universidad de Almeria, 04120 Almeria, Spain; 3 Museo Nacional de Ciencias Naturales, MNCN-CSIC, 28006 Madrid, Spain

**Keywords:** Gypsum Outcrops, Microbial Mats, *Fungi*, *Ascomycota*, *Basidiomycota*, *Mortierellomycota*, *Lecanicillium*, *Cladosporium*, *Mortierella*, *Podila*, Bats, Arthropods

## Abstract

The Gypsum Karst of Sorbas, Almeria, southeast Spain, includes a few caves whose entrances are open and allow the entry and roosting of numerous bats. Caves are characterized by their diversity of gypsum speleothems, such as stalactites, coralloids, gypsum crusts, etc. Colored biofilms can be observed on the walls of most caves, among which the Covadura and C3 caves were studied. The objective was to determine the influence that bat mycobiomes may have on the fungal communities of biofilms. The results indicate that the fungi retrieved from white and yellow biofilms in Covadura Cave (*Ascomycota*, *Mortierellomycota*, *Basidiomycota*) showed a wide diversity, depending on their location, and were highly influenced by the bat population, the guano and the arthropods that thrive in the guano, while C3 Cave was more strongly influenced by soil- and arthropod-related fungi (*Ascomycota*, *Mortierellomycota*), due to the absence of roosting bats.

**Supplementary Information:**

The online version contains supplementary material available at 10.1007/s00248-024-02395-y.

## Introduction

Caves are populated by a wide variety of microorganisms, including bacteria, fungi, algae, protozoa, and more, which thrive in both pristine and show caves. These microorganisms are found to be associated with biofilms that cover cave walls and sediments. Most caves present conspicuous biofilms, observable with the naked eye, in the form of round colonies, patches, or extensive microbial mats of different colors, generally white, yellow, and grey [1–5]. Furthermore, show caves subjected to artificial lighting exhibit wide phototrophic biofilms mainly composed of cyanobacteria and microalgae [6, 7]. The interest in the study of biofilms lies in their contribution to the physical and chemical deterioration of rocks and speleothems, which is of particular importance for the conservation of rock art that house many caves [1, 5, 7].

The case of Altamira Cave, Spain, is paradigmatic with respect to the colonization of its walls by different colored biofilms [1]. Jurado et al. [8] studied both biofilms and walls in this cave without visible microbial colonizations, detecting only bacterial DNA and no fungal DNA. The absence of fungi was further established by scanning electron microscopy [9]. This was later confirmed by González-Riancho Fernández [10]. This absence was attributed to the production of bioactive compounds with antifungal properties by bacteria that form the biofilms. In particular, many strains of *Crossiella, Streptomyces, Micromonospora, Nocardia, Pseudonocardia, Lechevalieria, Kibdelosporangium, Amycolatopsis, Saccharothrix,* among others, were isolated from these biofilms [11, 12]. For example, recently, two *Crossiella* strains isolated from Altamira white biofilms inhibited the growth of other bacteria and fungi. Their genomes showed an exclusive combination of gene clusters involved in the synthesis of lanthipeptides, lasso peptides, sactipeptides, furans, polyketide synthases, and non-ribosomal peptide synthetases [13].

Further studies corroborated the absence of fungi within cave biofilms. Martin-Pozas et al. [5] investigated yellow biofilms in Pindal Cave, Spain, applying specific CARD-FISH probes targeting *Ascomycota* and *Basidiomycota,* with negative results. Additionally, sequence analysis using fungal primers did not detect any fungal sequences, concluding that fungi were not present in the yellow biofilms. However, other authors found fungi in cave biofilms from different countries [7, 14, 15]. It should be noted that fungi are common in cave air, as well as in excrements, guano, and animal cadavers frequently found in caves and sediments [16, 17].

Many cave fungi have been related to arthropods and bats [12, 18–21], while others appear to be specific to cave ecosystems [22, 23]. Although considerable attention has been directed towards fungi in limestone and volcanic caves [2, 24–26], studies on gypsum caves are scarce, if not lacking.

Cunningham et al. [27] found in Lechuguilla, a gypsum cave in New Mexico, patches of fungi on deposits, and aspergilli associated with Fe-, Mn- and S-rich encrustations. Cacchio et al. [28] investigated the involvement of bacteria in the formation of speleothems in the gypsum cave Grave Grubbo, Italy. Mihajlovski et al. [29] studied the fungal diversity in gypsum efflorescences in a French cave and described the abundant presence of *Isaria fumosorosea* and *Engyodontium album*, two well-known entomopathogens [19].

Some gypsum karsts are important in Western Europe because they are excellent examples (geoheritage sites) of emerged outcrops of selenitic gypsum, which formerly corresponded to different peripheral basins around the Mediterranean with a diachronous precipitation of evaporite deposits during the Messinian age. These karstified gypsum outcrops are mainly located in the Apennines and Sicily, Italy [30], while in Spain the Gypsum Karst of Sorbas in Almeria is notable [31]. The geology, geomorphology, hydrogeology, and microclimatology of this last karst have been extensively studied in the last two decades [32–35]; however, the microbial communities of their gypsum caves are unknown.

In the gypsum karst of Sorbas, Covadura Cave harbored an abundant bat population, although this has decreased considerably in recent years, with only isolated individuals currently detected in the galleries sampled in this study. In C3 Cave there is an absence of roosting bats. Altamira and Pindal caves, located in northern Spain, do not have bats in their confined and close environments. Nonetheless, all the four caves show abundant white and yellow biofilms composed of bacteria, but no fungi were retrieved from the Altamira and Pindal caves [5, 10, 36], and unpublished data. Here, we study the fungal diversity in the biofilms that cover the walls of two gypsum caves to determine whether bat microbiomes indeed have influence on the wall biofilms.

## Material and Methods

### The Gypsum Karst of Sorbas

**Fig. 1:**
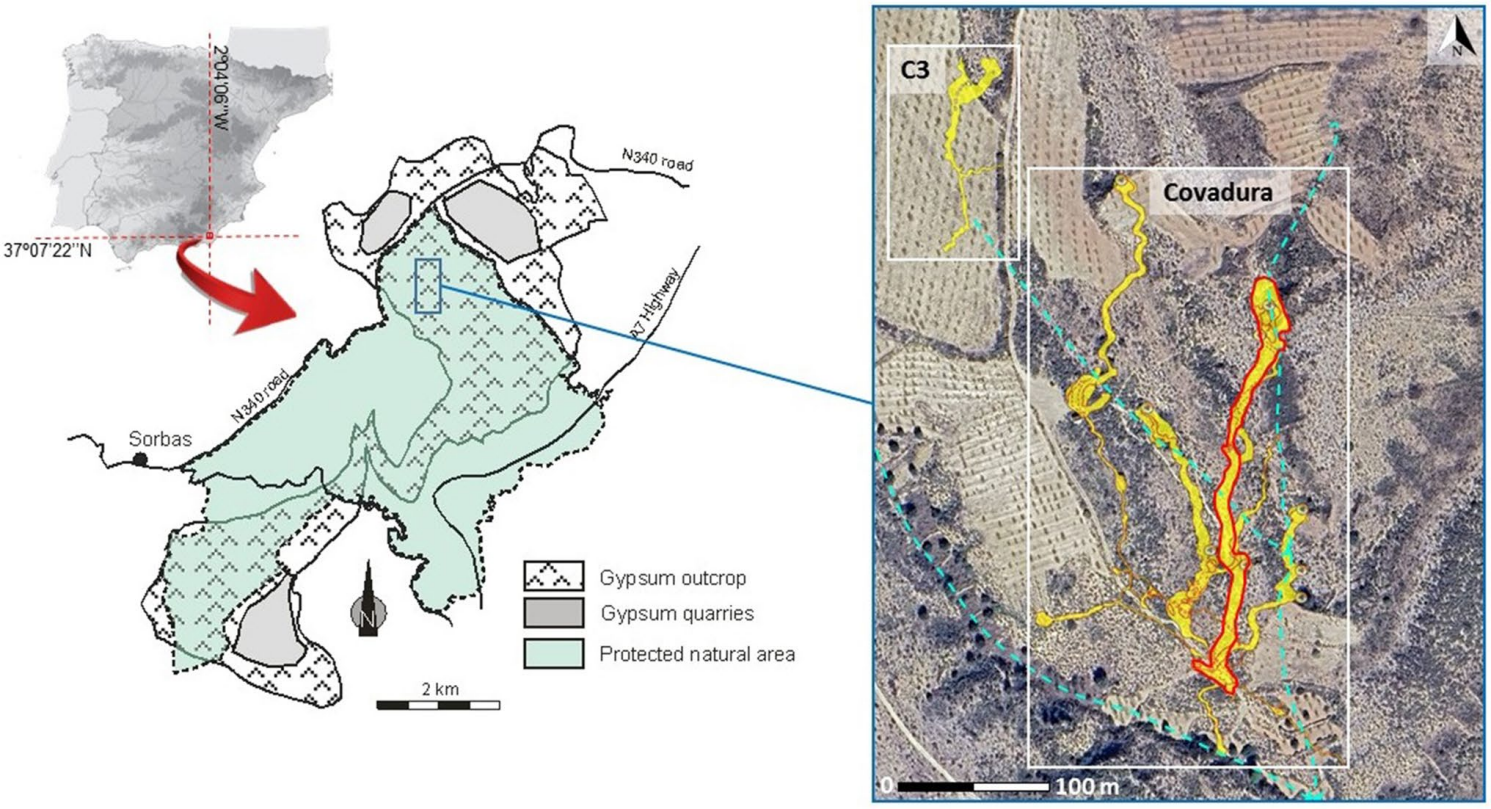
Location of the Gypsum Karst of Sorbas (southern Spain) and spatial distribution of the galleries of the Covadura and C3 caves (yellow areas) in relation to the external topography. The dashed blue lines show the drainage network on the surface. The yellow areas highlighted by a red line correspond to the galleries studied in the Covadura Cave

The Gypsum Karst of Sorbas, is located in the province of Almeria, southeast Spain (Fig. 1). This is formed by significant gypsiferous Messinian evaporates within a 120 m thick cyclic sequence formed by alternating gypsum and marly units, and many caves amounting to 100 km of passages, including the six most relevant caves for their extension and representativeness in the local karst hydrogeology (Covadura Cave, C3 Cave, Gypsum Cave, Water Cave, Treasure Cave and GEP Complex) [31].

**Fig. 2:**
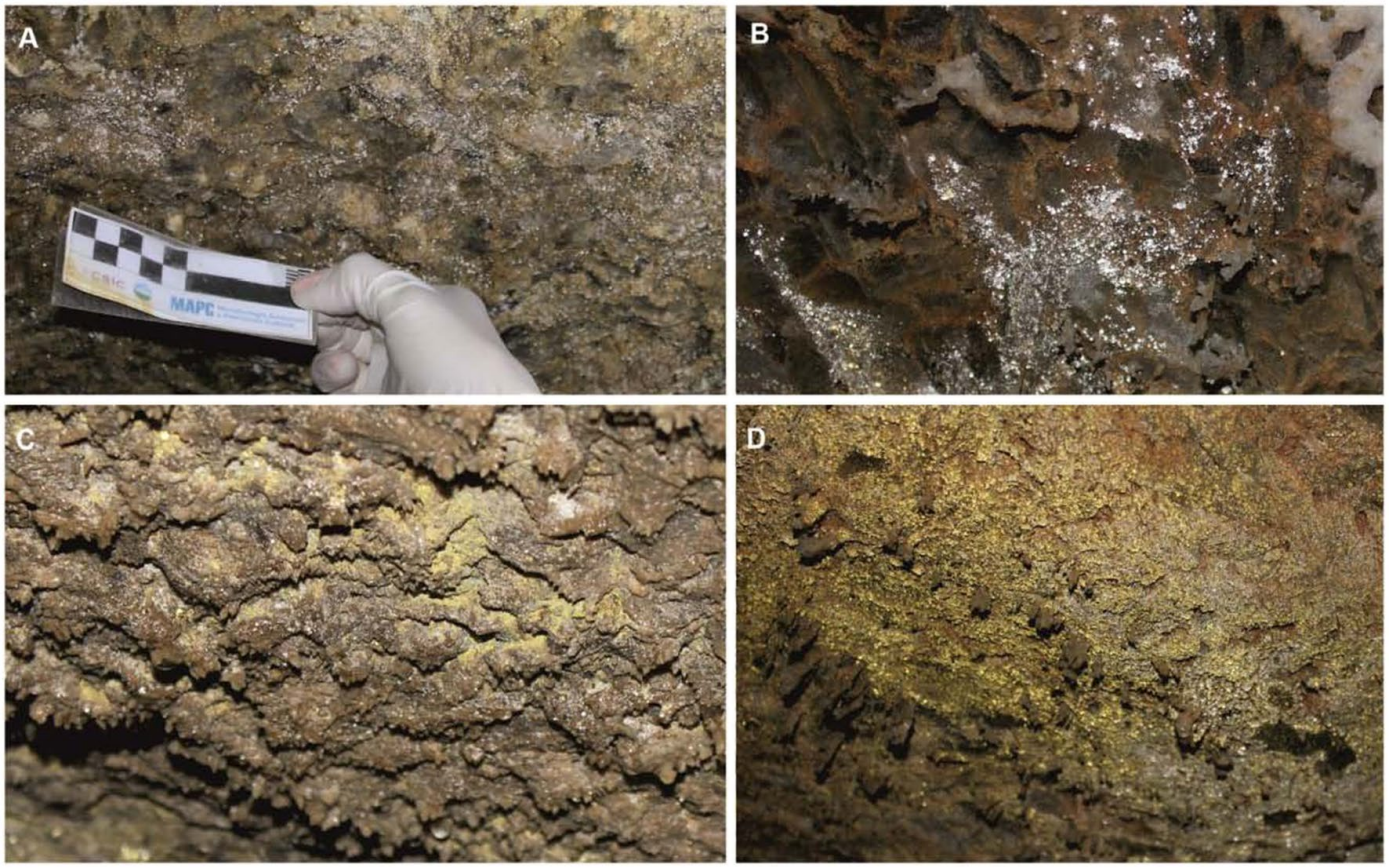
Colored biofilms on the walls of Covadura and C3 caves. **A**. White biofilm in Covadura Cave; **B**. White biofilm in C3 Cave; **C**. Yellow biofilm in Covadura Cave; **D**. Yellow biofilm in C3 Cave

The Covadura Cave is 120 m deep and 275 m long in the studied area, although the system has more than 4 km of galleries. The C3 Cave, at some 200 m from the Covadura Cave, is only about 3 m deep and has a length of 150 m [34]. A diversity of gypsum speleothems, such as stalactites, coralloids, gypsum crusts, etc. have been described in these caves, as well as colored biofilms (Fig. 2).

The Covadura Cave harbored a bat population of more than 5,000 bats, composed of the species *Rhinolophus ferrumequinum, Rhinolophus hipposideros*, and *Miniopterus schreibersii*. *Rhinolophus euryale* has occasionally been found [37]. Access to the caves is forbidden from October to March to protect the bats during the hibernation period. In Covadura Cave there has been some activation of the watercourse and partial flooding washed away guano; however, the area of wall biofilms has not been affected and may have retained previously existing fungal populations. However, through visits and sampling carried out in recent years, the disappearance of guano in the cave has been confirmed. In Cave C3, located about 200 m from Covadura Cave, there have never been bat colonies.

The climate in the Sorbas area is semi-arid Mediterranean, with a mean annual temperature and rainfall of 17 °C and 274 mm, respectively. Estimated annual potential evapotranspiration is nearly five times the mean annual precipitation, and about 80% annual rainfall during low-frequency rainstorm events, usually in the autumn. The vegetation comprises scattered shrubs and dwarf shrubs, as well as some crass perennial plants are also common. The cover of perennial vegetation is below 40 % at both study sites and is mainly made up of tussock grass. Plant interspaces are usually colonized by well-developed biocrusts dominated by lichens.

### Preparation and Characterization of Samples by Scanning Electron Microscopy (SEM)

Biofilms were collected and transported at 4°C without separating them from the mineral support on which they were growing. Sample preparation for SEM analysis was carried out as described by Martin-Pozas [5]. Biofilm structures were examined under high and low vacuum conditions using an environmental scanning electron microscope Inspect (FEI, USA).

### Sampling Method

In 2010 and 2022, nine white and yellow biofilm samples on the gypsum walls were collected in Covadura Cave, while only two biofilm samples from the C3 Cave were sampled in 2022 (Supplementary Figure S1). Biofilm samples were collected using sterile scalpels and preserved in Lifeguard preservation solution (Qiagen, Hilden, Germany) until their arrival in the laboratory, where they were stored at -80°C.

### DNA Extraction, Sequencing and Data Processing

Genomic DNA extraction was performed using the FastDNA SPIN Kit for Soil (MP Biomedicals, Illkirch, France). DNA concentrations were quantified using a Qubit 2.0 fluorometer (Invitrogen, Carlsbad, CA, USA). High-throughput sequencing of the extracted DNA was carried out by FISABIO Institute (Valencia). The intergenic transcribed spacers (ITS) of fungal organisms were amplified according to Toju *et al.* [38]. The primer sequences used were ITS3_KYO2 (GATGAAGAACGYAGYRAA) and ITS4_KYO1 (TCCTCCGCTTWTTGWTWTGC), using Illumina MiSeq and 2 × 300 paired end sequencing. Sequence data have been performed using the Qiime2 pipeline [39]. Metataxonomy analysis was performed using some of the qiime2 plugins. Denoising, joining of the paired-ends, and depletion of the chimera was performed starting from data from the paired ends using DADA2 pipeline [40]. Taxonomic affiliations were assigned using the Naive Bayesian classifier integrated in the quiime2 plugins. The database used for taxonomic assignment was UNITE version 10.0 [41]. All computations and statistics were performed within the R Statistics environment [42]. Taxonomic data obtained in QIIME2 were imported into R and analyzed with the phyloseq package. From these taxonomic data, ecological roles were assigned using the FUNGuildR tool and the FUNGuild database [43]. To assess ecological alpha diversity within each sample, we used non-phylogenetic diversity indices such as Chao1, Shannon, and Simpson. These common metrics were calculated using raw species-level data with R packages phyloseq and vegan. The plots and heatmap were built using the R packages ggplot 2 and pheatmap.

### Deposit of Sequences

The raw reads were deposited into the NCBI Sequence Read Archive (SRA) database under the project number PRJNA1103123.

## Results

**Fig. 3:**
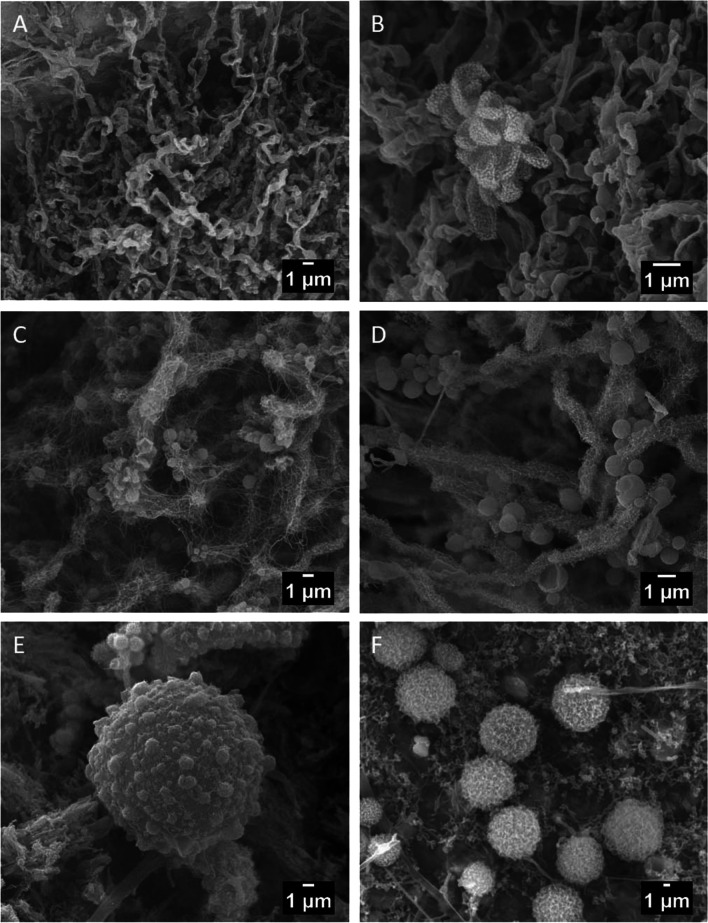
SEM images of biofilms found on the walls of the Covadura and C3 caves. A network of bacterial structures with characteristics of *Actinomycetota*, the most abundant phylum, is shown in A-D images. A. White biofilm in Covadura Cave; B. White biofilm in C3 Cave; C. Yellow biofilm in Covadura Cave; D. Yellow biofilm in C3 Cave. E – F. Long smooth filaments (1.4 μm width) and spores (10-14 μm diameter) of fungi found in Covadura Cave

Biofilms are mainly composed of bacteria, but fungal spores were also evident (Fig. 3). SEM images of these biofilms were similar to those obtained in the biofilms from Castañar Cave, with a high abundance of bacteria and fungi [25, 44]. The bacterial composition of the biofilms will be discussed in a further work, but we can anticipate that white and yellow biofilms in these gypsum caves are mainly composed of bacteria with a relatively high abundance of two main phyla: *Actinobacteriota* and *Pseudomonadota*, followed by *Acidobacteriota* and *Planctomycetota*.

In the study of fungi, a total of 2,515,260 high-quality sequence reads were obtained, ranging from 8,652 to 309,474 from the white and yellow biofilms (Supplementary Table S1). In total, 422 amplicon sequence variants (ASVs) were assigned based on 99% sequence similarity. According to diversity indices, samples with the highest Chao1, Shannon, and Simpson values are found in the white and yellow biofilms from Covadura Cave, indicating greater species diversity and more uniform distribution of relative species abundance compared to samples from Cave C3 (Supplementary Table S1 and Figure S2). In summary, Covadura Cave exhibits greater species richness and diversity than C3 Cave, and specifically, the yellow biofilms exhibit higher species diversity, particularly those from 2010.

**Fig. 4:**
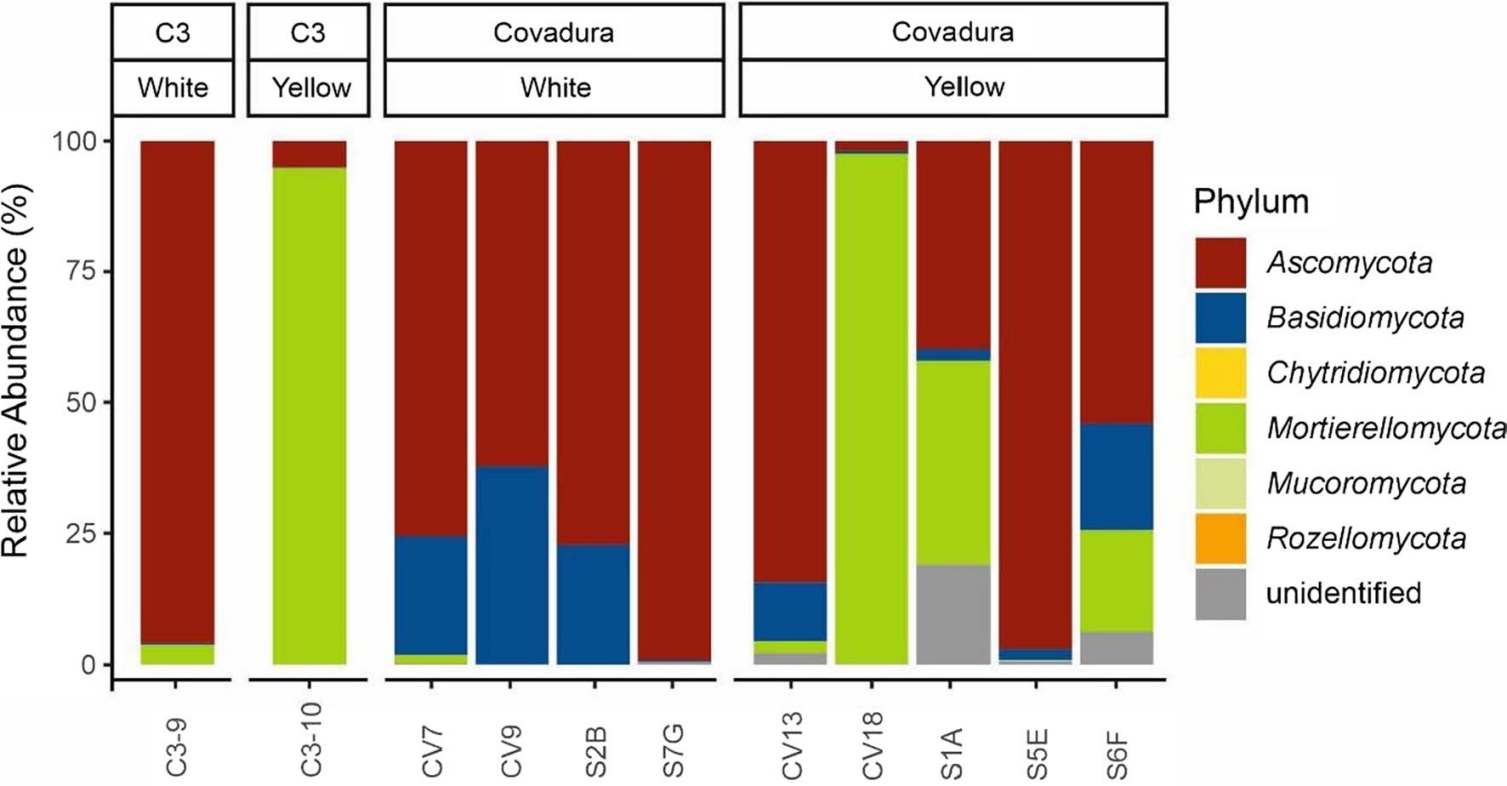
Fungal community composition of biofilms at the phylum level in Covadura and C3 caves

In the white biofilms collected in the Covadura Cave, *Ascomycota* (relative abundance 62.2% – 99.1%) and *Basidiomycota* (0.4% – 37.7%) were the most abundant phyla. However, yellow biofilms showed a different and variable pattern, with higher abundances of *Mortierellomycota* in three samples (19.2% – 97.4%), *Ascomycota* in four samples (39.8% – 97.0%) and *Basidiomycota* in the other two samples (11.0% – 20.4%). Additionally, a yellow biofilm sample presented 19.0% relative abundance of a phylum at an unclassified taxonomic rank. Negligible or no relative abundances were observed for other phyla, including unidentified sequences (Fig. 4). In general, a decrease in *Ascomycota* relative abundances was detected in white biofilms in 2022 (75.6 – 62.2%) compared to levels recorded in 2010 (77.3 – 99.1%), although no consistent pattern was observed in yellow biofilms, with variable relative abundances between samples. This lack of consistency was also evident in the abundances of *Basidiomycota* and *Mortierellomycota*.

In C3 Cave, the fungal community in the white biofilm was predominantly composed of *Ascomycota*, representing 95.9% of the relative abundance, followed by *Mortierellomycota* (3.8%). The yellow biofilm exhibited a reverse trend, with *Mortierellomycota* comprising 94.8% of the community and *Ascomycota* representing around 5.0%.

**Fig. 5:**
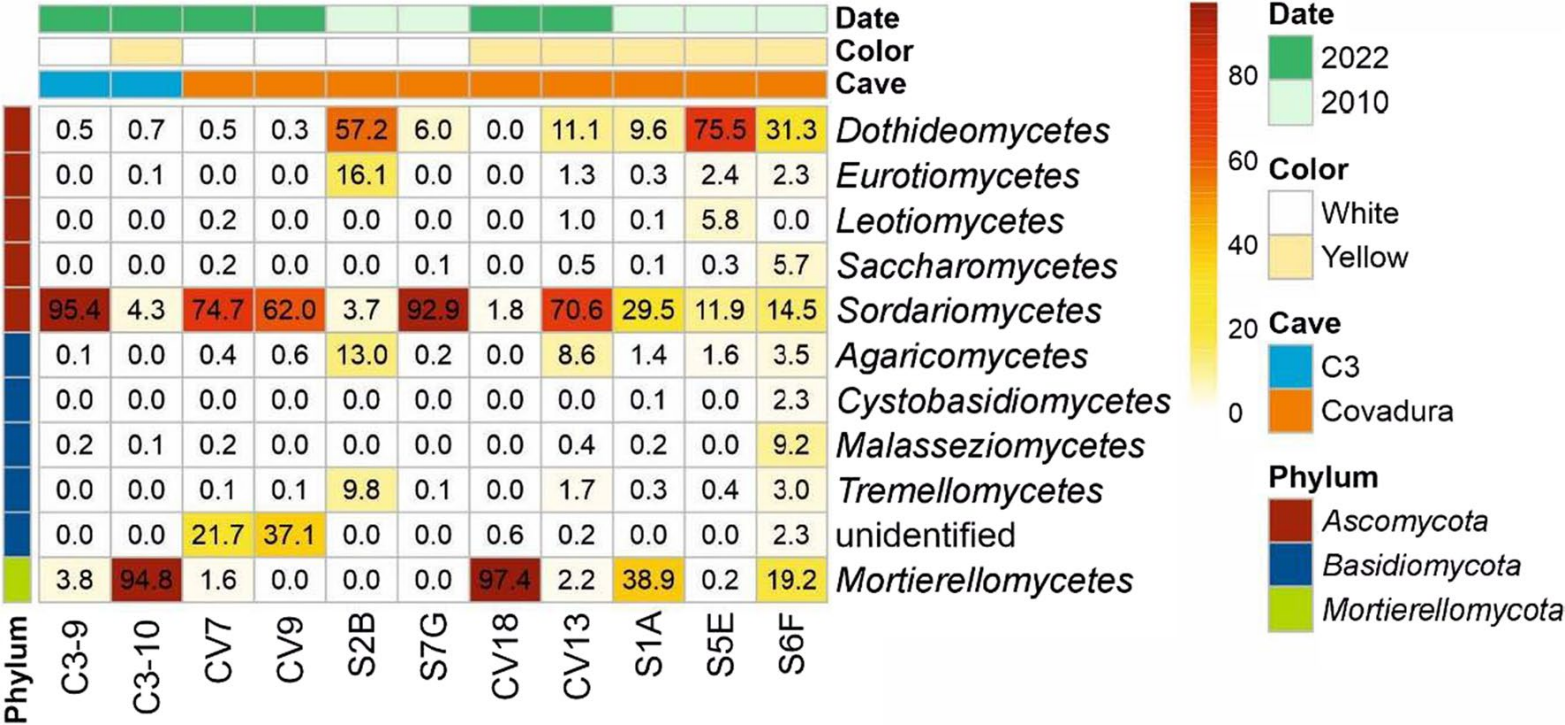
Heat map of fungal classes in biofilms from Covadura and C3 caves. Only classes with relative abundances >1% are represented

In all samples, the most abundant classes were *Sordariomycetes, Mortierellomycetes*, and *Dothideomycetes. Sordariomycetes* were represented in all samples from the two caves, although with variable abundance depending on the samples (1.8% – 95.4%) (Fig. 5). *Mortierellomycetes* were relatively abundant in the yellow biofilms of Covadura Cave (0.2% – 97.4%), and in C3 Cave (94.8%), but only appeared in a sample of white biofilms from Covadura Cave (1.6%) and in C3 (3.8%). *Dothideomycetes* were more abundant in the yellow biofilms collected in the Covadura Cave in 2010 (9.6% – 75.5%) than in 2022 (0% – 11.1%) as well as for the white biofilms (6.0% – 57.2% in 2010 vs 0.3% – 0.5% in 2022). An unidentified *Basidiomycota* taxonomic class presented relative abundances of 21.7% and 37.1% in the two white biofilms from Covadura Cave collected in 2022.

*Eurotiomycetes* and *Agaricomycetes* were relatively abundant in only one sample of Covadura Cave white biofilms collected in 2010 (16.1% and 13.0%, respectively)*.* In the white biofilms samples collected in 2022 in the Covadura Cave, *Eurotiomycetes* were absent and *Agaricomycetes* showed low abundances (0.4 – 0.6%). In the yellow biofilms of Covadura Cave *Eurotiomycetes* reached low abundances in 2022 (0% – 1.3%) and in 2010 (0.3% – 2.4%) and *Agaricomycetes* slightly higher in 2022 (0% – 8.6%) than in 2010 (1.4% – 3.5%). In the C3 Cave, the abundances of these two classes were negligible. Relative abundances for other classes were missing or negligible, except for the 2010 white (*Tremellomycetes* 9.8%) and yellow biofilm samples (*Leotiomycetes* 5.8%, *Saccharomycetes* 5.7% and *Malasseziomycetes* 9.2%).

**Fig. 6:**
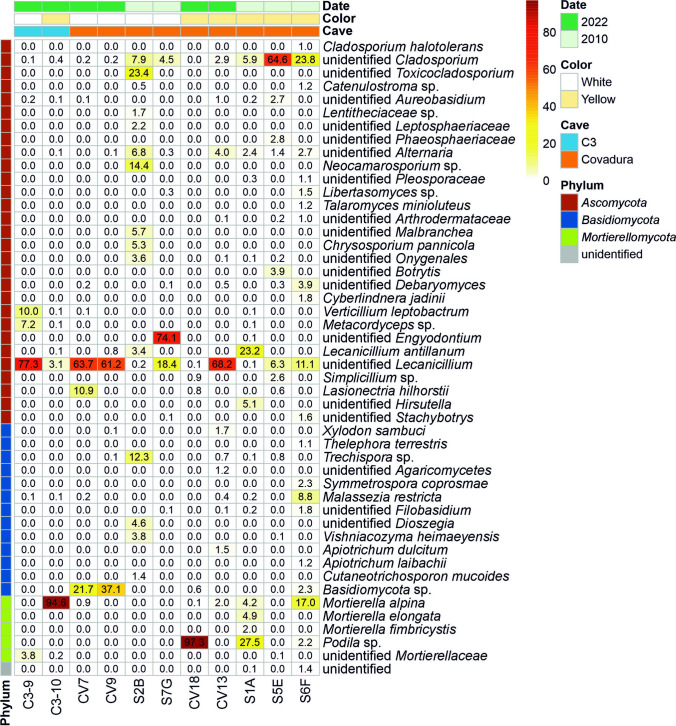
Heat map of fungal species in biofilms from Covadura and C3 caves. Only ASVs at Species level with relative abundances >1% are shown

More than 100 fungal genera were identified, with an additional forty-six genera remaining unidentified in the Covadura and C3 caves. Significant differences were observed between white and yellow biofilms on the abundance of the genera (Fig. 6). In Covadura Cave, several genera presented relative abundances above 10% in different biofilms, but the distribution between white and yellow biofilms was different. The 2010 white biofilms were characterized by the abundant occurrence (>10%) of *Engyodontium, Toxicocladosporium, Lecanicillium, Neocamarosporium* and *Trechispora*, and in the 2022 white biofilms of *Lecanicillium, Lasionectria* and an unidentified *Basidiomycota.* The yellow biofilms showed a high abundance of *Lecanicillium, Cladosporium, Podila,* and *Mortierella* in the 2010 samples, and of *Lecanicillium* and *Podila* in the 2022 samples. Other genera also well represented (5-10%) included *Alternaria, Malbranchea* and *Chrysosporium* in white biofilms and *Malassezia* and *Hirsutella* in yellow biofilms. In the C3 Cave, only *Lecanicillium* and *Verticillium* were important (>10%) in the white biofilms and *Mortierella* in the yellow ones. *Metacordyceps* (7.2%) was identified in the white biofilms.

The most abundant and common genus in all samples was *Lecanicillium*, an insect pathogen, both in Covadura white and yellow biofilms, as well as in C3 white and yellow biofilms. *Lecanicillium antillanum* was relatively abundant (23.2%) in a yellow biofilm from 2010.

Other well-known entomopathogenic fungal genera are *Engyodontium (=Beauveria), Hirsutella, Simplicillium*, *Metacordyceps*, etc., also found in Covadura biofilms. *Engyodontium* was only abundant (74.1%) in a white biofilm collected in 2010, while *Hirsutella* (5.1%) and *Simplicillium* (2.6%) were found in yellow biofilms of the same year.

The *Mortierellaceae* include the genus *Mortierella*, *Linnemannia,* and *Podila* among others. *M. alpina* reached a relative abundance of 94.6% in the yellow biofilm of the C3 Cave and 17.0% in a 2010 yellow biofilm from the Covadura Cave. *M. elongata* was identified in a 2010 yellow biofilm (4.9%).

The genus *Podila* was established in 2020 to accommodate some *Mortierella* species that are difficult to identify by ITS sequences [45]. *Podila* was represented in yellow biofilms with 97.3% relative abundance in a yellow biofilm from 2022 and 27.5% in another from 2010.

The genus *Cladosporium* was relatively abundant in three 2010 yellow Covadura biofilms (5.9 –64.6%), less abundant in 2010 white biofilms (4.5 – 7.9%), and largely absent in most 2022 samples. Its abundance in the C3 Cave was also insignificant (0.0 – 0.4%), *Cladosporium halotolerans* was only identified in a yellow biofilm from 2010 (1.0%).

The species *Verticillium leptobactrum* (= *Leptobacillium leptobactrum*), a recognized entomopathogenic fungus, was identified in the white biofilm of C3 Cave (10.0% relative abundance) and 0.1% in a few Covadura and C3 biofilms. *Metacordyceps* reached 7.2% in the white biofilm of C3 Cave and 0.1% in a yellow Covadura biofilm.

Abundant genera, but only present in a unique sample of a Covadura 2010 white biofilm, were *Toxicocladosporium*, and *Neocamarosporium*. These two genera and a few others such as *Catenulostroma, Libertasomyces, Xylodon, Symmetrospora,* and *Thelephora*, were recovered in low abundances in the biofilms.

**Fig. 7:**
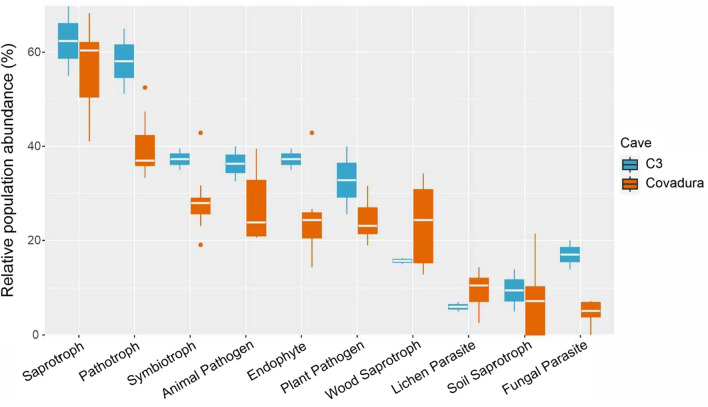
Functional assignments based on FUNGuild database of all functions of biofilms from Covadura and C3 caves

A detailed classification of fungal traits is presented in Fig. 7. There, it can be observed that the abundances of saprotrophs and pathotrophs were higher than 60% of the total relative abundances in C3, while the abundances of saprotrophs were also high in Covadura Cave, but the pathotrophs decreased to 40%. Other important traits were wood saprotroph and animal pathogen.

## Discussion

Many studies reported the presence of fungi in caves, particularly in phototrophic biofilms. Predominantly, these fungi belong to the phyla *Ascomycota, Zygomycota* and *Basidiomycota* [7, 14, 46]. The dominant fungal phyla in the Covadura and C3 gypsum caves were *Ascomycota, Mortierellomycota*, and *Basidiomycota.* This is in agreement with data from other authors in karstic caves [25, 46].

The most abundant classes in the two caves were *Sordariomycetes, Mortierellomycetes*, and *Dothideomycetes.* Similar abundances of these three classes have been reported in a few caves from different countries and rock surfaces [47–50].

The *Sordariomycetes* are represented by the genera *Lecanicillium*, *Engyodontium,* and *Lasionectria,* with abundances over 10% in at least one of the samples. The genus *Lecanicillium* comprises insect pathogens and also parasitizes other arthropods, fungi, and plants [51]. Different species of *Lecanicillium* were reported in Spanish and other European caves with an abundance of arthropods [17, 18, 24, 52, 53]. However, only one species of *Lecanicillium* was identified, *L. antillanum* in the 2010 Covadura white and yellow biofilms and the C3 yellow biofilm.

The caves in the Gypsum Karst of Sorbas harbor a wide diversity of arthropods, along with bats. Indeed, many authors have recorded the occurrence of numerous arthropod taxa in these caves, including members of the orders *Araneida, Anactinotrichida*, *Opilionida*, *Pseudoescorpionida, Isopoda, Quilopoda, Collembola, Psocoptera, Thysanoptera, Thysanura, Hemiptera, Coleoptera, Siphonaptera, Diptera, Diplura, Orthoptera*, *Trichoptera*, among others [54, 55]. Most of these orders and others have been reported in bat guano in different geographical locations [56, 57]. Therefore, the abundant occurrence of *Lecanicillium* is probably linked to cave arthropods and bat guano.

The *Mortierellomycetes*, *(Mortierella*, *Linnemannia, Podila*, etc.), are saprobic organisms with a widespread distribution in a wide range of habitats [45]. Among all *Mortierella* species, the most commonly recovered in caves were *M. alpina* and *M. elongata* (=*Linnemannia elongata*) [24, 25, 59]. Many species of the genus *Mortierella* have been isolated from bats, dung samples collected in caves and mines, wings and bat carcasses, in addition to soils where they are abundant [16, 58–60]. *Podila* species are frequent in soils, compost and dung [45].

In addition to *Mortierella,* a large number of genera found in the biofilms of Covadura Cave have previously been recovered from bat guano. These include *Lecanicillium, Cladosporium, Alternaria, Aureobasidium, Botrytis, Simplicillium, Debaryomyces, Talaromyces, Chrysosporium*, *Apiotrichum*, and *Cutaneotrichosporon*, while only *Lecanicillium, Cladosporium, Alternaria*, and *Aureobasidium* were found in C3 Cave [21, 24, 61–63]. Furthermore, most of these genera and others (*Filobasidium, Malassezia, Vishniacozyma, Wallemia, Comoclasthris, Cephalotrichum, Lasionectria, Solicozyma, Acremonium,* etc.) were isolated from bat mycobiomes [58, 64].

The *Dothideomycetes* are represented by the genera *Cladosporium*, *Toxicocladosporium*, and *Neocamarosporium* with significant abundances. *Cladosporium halotolerans,* the most frequently isolated indoor species [65], was found in the two gypsum caves, and previously in other Spanish caves [17]. Other species of *Cladosporium* are ubiquitous plant pathogens and were also isolated from caves [17, 66]. *Toxicocladosporium* was detected in biofilms from stones [67], and plants [68], as well as *Neocamarosporium* [69, 70].

Important ecological traits observed in the genera found in the Covadura and C3 biofilms were the chitinolytic and keratinolytic activities. Chitinolytic activity is related to the occurrence of insectivorous bats and chitin remains in the guano. Chitinolytic fungal genera were identified in both caves but with a higher diversity in Covadura (*Cladosporium, Alternaria, Verticillium, Aureobasidium, Acremonium, Trichoderma, Talaromyces, Botrytis, Stachybotrys*, and *Mortierella* than in C3 (*Verticillium, Aureobasidium, Acremonium, Mortierella*).

Keratinolysis is common in members of the order *Onygenales* (*Malbranchea, Chrysosporium*) and *Eurotiales* (*Cladosporium*, *Talaromyces*). Another fungal group of interest in Covadura Cave is the class *Tremellomycetes* that includes the basidiomycetous yeasts *Filobasidium, Apiotrichum, Vishniacozyma, Cutaneotrichosporon,* and *Dioszegia*. These genera have been associated with bats [64, 71]. In the C3 Cave only *Vishniacozyma* (=*Cryptococcus*) was identified. Members of this genus are common in soils and plants [72, 73].

Of no less interest is the increased abundance of a few genera (*Lecanicillium, Lasionectria,* unidentified *Basidiomycota*) in the Covadura 2022 sampling of white biofilms and of *Lecanicillium* and *Podila* in the yellow biofilms. The decrease in abundance in 2022 of a few genera that were abundant in the 2010 yellow biofilms (*Engyodontium, Mortierella*) is noteworthy. These changes can be attributed to fluctuations in arthropod/bat populations and/or severe droughts in recent years.

When comparing the Covadura and C3 cave biofilms with those of the Altamira and Pindal limestone caves, the strongest difference was the absence of fungi in the limestone biofilms [5, 8, 10]. In the limestone caves no bat roosts were found. The entrances to the gypsum caves are open, allowing the entry and roosting of bats. Therefore, the high number of fungal genera identified in the bat microbiome by a few authors [45, 64, 74], and their finding in cave biofilms, are not surprising. Furthermore, Hathaway et al. [58] reported that bat microbiomes have an influence on the cave wall microbiome, and Kokurewicz et al. [75] stated that the number of bats was a key factor determining the occurrence of fungal spores at the hibernation site.

There is a general consensus on the influence of arthropods, bats and guano on cave fungi. Yoder et al. [76] reported the presence of entomopathogenic fungi associated to cave spiders. Vanderwolf et al. [77] studied the fungi in three caves, one populated by bats and the other two without bats. They concluded that caves with active bat hibernacula supported bat-related fungi, while caves without bats, but abundant arthropods, contained entomopathogenic fungal genera. The conclusions of this and other papers [58, 64, 74–76] were confirmed in this study on biofilms from gypsum caves.

In summary, the fungi recovered from the different white and yellow biofilms in Covadura Cave showed a wide diversity, with differences depending on their location and year of sampling, and were highly influenced by the presence of bats, guano, and arthropods that thrive in the guano. In the C3 Cave the occurrence of bat-related fungi was low, probably due to the absence of roosting bats, with the cave environment dominated by soil- and arthropod-related fungi.

### Supplementary Information

Supplementary Material 1: (PDF 433 kb)

## Data Availability

The gene sequences and accompanying metadata were deposited in the Sequence Read Archive (SRA) of NCBI under the project number PRJNA1103123.
