# Supplementary material for: Gypsum Cave Biofilm Communities are Strongly Influenced by Bat- And Arthropod-Related Fungi
Source: Microb Ecol. 2024 Jun 3;87(1):80. doi: 10.1007/s00248-024-02395-y (PMC11147836; doi:10.1007/s00248-024-02395-y)
Supplement: Supplementary file 1 — (PDF 433 kb) [file 248_2024_2395_MOESM1_ESM.pdf]

SUPPLEMENTARY INORMATION

GYPSUM CAVE BIOFILM COMMUNITIES ARE STRONGLY INFLUENCED BY BAT- AND ARTHROPOD-RELATED FUNGI

Valme Jurado[1], Tamara Martin-Pozas[2], Angel Fernandez-Cortes[2], Jose Maria Calaforra[2], Sergio Sanchez-Moral[3], Cesareo Saiz-Jimenez[1]

[1] Instituto de Recursos Naturales y Agrobiologia, IRNAS-CSIC, 41012 Sevilla, Spain

[2] Departamento de Biologia y Geologia, Universidad de Almeria, 04120 Almeria, Spain

[3] Museo Nacional de Ciencias Naturales, MNCN-CSIC, 28006 Madrid, Spain

Table S1. Location and estimation of α-diversity indices for each sample from Covadura and C3 Caves

Cave	Biofilm	Year	Samples	Reads	ASVs*	Chao1	Shannon	Simpson
Covadura	White	2022	CV7	48,888	39	27	1.08	0.54
			CV9	26,364	21	16	0.77	0.49
		2010	S2B	1,692	26	22	2.48	0.89
			S7G	18,433	38	31	0.87	0.42
C3		2022	C3-9	29,400	20	19	0.84	0.39
Covadura	Yellow	2022	CV13	32,507	111	75	1.76	0.53
			CV18	79,677	14	10	0.16	0.05
		2010	S1A	53,003	105	67	2.16	0.82
			S5E	38,086	101	81	1.83	0.57
			S6F	37,872	58	37	2.68	0.89
Covadura		2022	C3-10	81,491	43	32	0.31	0.10

*: Amplicon Sequence Variant

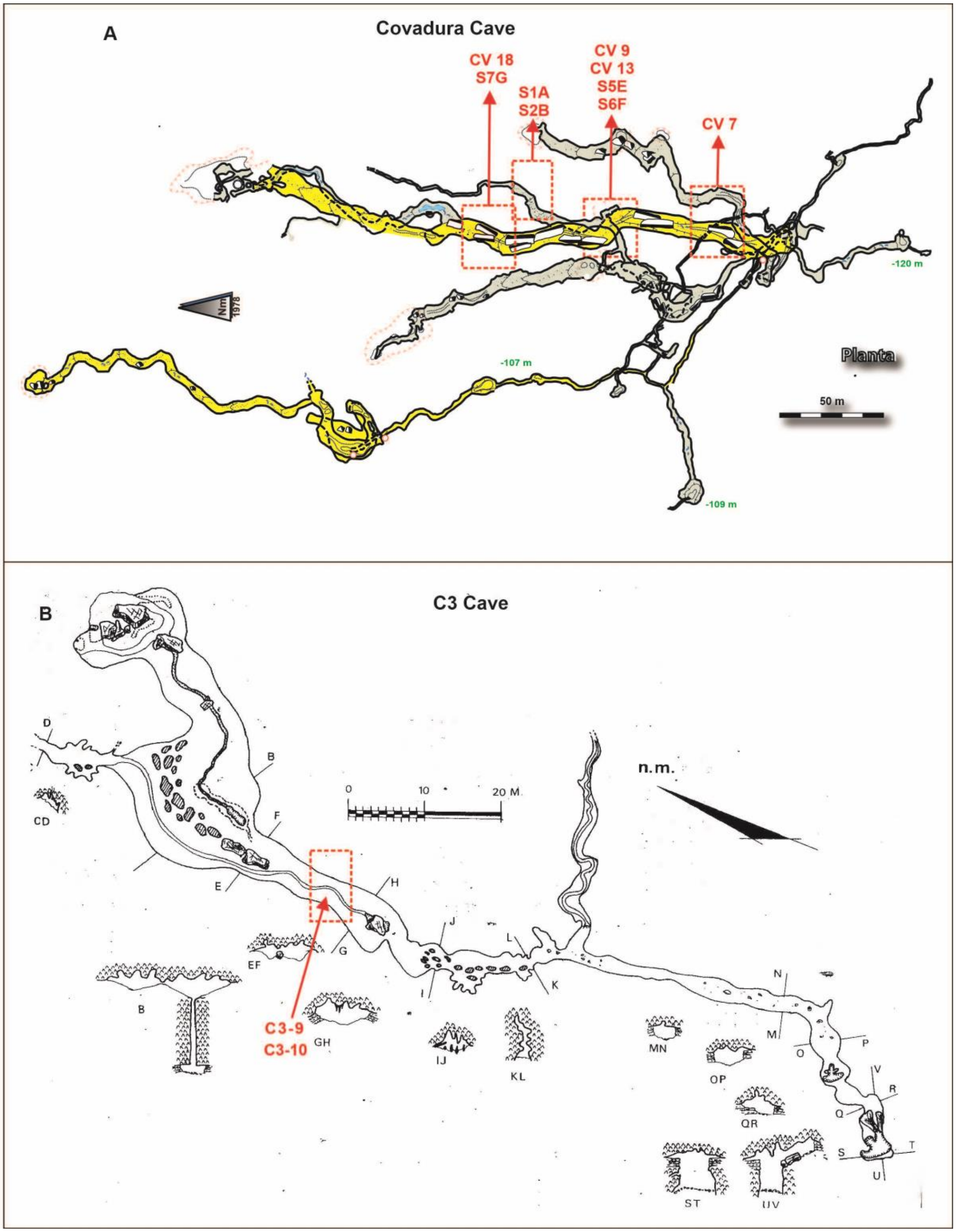

Figure S1. Map of Covadura and C3 caves with the location of sampling points in 2010 (S1A, S2B, S5E, S6F, S7G) and 2022 (CV7, CV9, CV13, CV18, C3-9, C3-10).

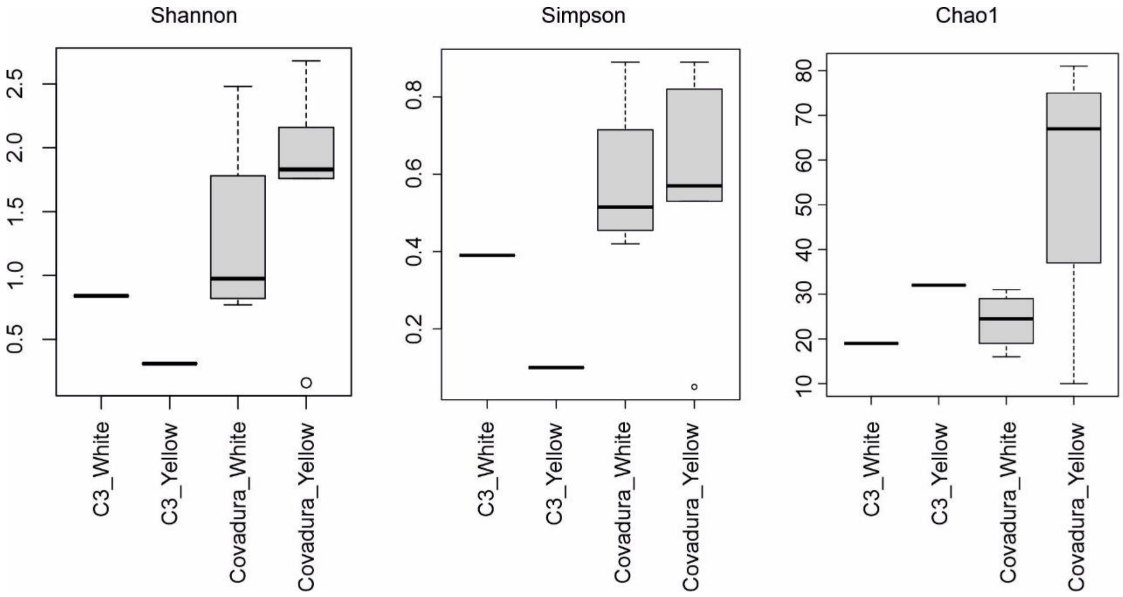

Figure S2. α-Diversity indices for each sample from Covadura and C3 Caves.
